# Heat stress and immune response phenotype affect DNA methylation in blood mononuclear cells from Holstein dairy cows

**DOI:** 10.1038/s41598-021-89951-5

**Published:** 2021-05-31

**Authors:** A. M. Livernois, B. A. Mallard, S. L. Cartwright, A. Cánovas

**Affiliations:** 1 Deptartment of Pathobiology, Ontario Veterinary College, University of Guelph, Guelph, ON Canada; 2 Centre for Genetic Improvement of Livestock, Department of Animal Biosciences, University of Guelph, Guelph, ON Canada

**Keywords:** Epigenomics, Immunogenetics, Sequencing, Immunology

## Abstract

Heat stress negatively affects health and production in cows. Examining the cellular response to heat stress could reveal underlying protective molecular mechanisms associated with superior resilience and ultimately enable selection for more resilient cattle. This type of investigation is increasingly important as future predictions for the patterns of heat waves point to increases in frequency, severity, and duration. Cows identified as high immune responders based on High Immune Response technology (HIR) have lower disease occurrence compared to their average and low immune responder herd-mates. In this study, our goal was to identify epigenetic differences between high and low immune responder cows in response to heat stress. We examined genome-wide DNA methylation of blood mononuclear cells (BMCs) isolated from high and low cows, before and after in vitro heat stress. We identified differential methylation of promoter regions associated with a variety of biological processes including immune function, stress response, apoptosis, and cell signalling. The specific differentially methylated promoter regions differed between samples from high and low cows, and results revealed pathways associated with cellular protection during heat stress.

## Introduction

The changing climate is impacting livestock health and welfare through increased environmental temperatures and drought^1–3^. Ambient temperature and humidity above a comfort threshold result in heat stress in cattle^4^ with signs including discomfort, increased respiration rate, dehydration, changes in cardiac function, and even death^5^. To ensure cell survival and growth during heat stress, eukaryotic organisms respond by activating transcription of genes that protect against damage and cell death. Heat shock proteins (HSPs) play a major role in the response to heat stress and also other types of environmental stress^6^. These molecular chaperones can inhibit apoptosis and facilitate cell proliferation and protein re-folding that preserve protein structure and transport^7,8^. As such, elevated expression of HSPs is a desired response to heat stress^9–11^.

The effects of stress on immune function have been well studied, showing immune activation if stress is brief and intense (acute stress) or immune suppression if stress is persistent over a period of time (chronic stress)^12–15^. There is evidence of associations between the immune response and the response to other external stressors, such as heat stress, in ruminants. For example, heat stress has been shown to increase blood cortisol concentration which inhibits the production of some cytokines^16^. Transcriptomics studies have consistently shown differential expression of genes involved in immune response between heat stressed and non-heat stressed animals^17–22^. Additionally, multiple studies have found that exposure of bovine blood mononuclear cells (BMCs) to short and severe heat stress reduced the response to mitogen stimulation or decreased the number of viable cells^23–25^.

The high immune response (HIR) technology identifies cows with balanced and robust cell-mediated and antibody-mediated immune responses compared with their herd mates^26^. High-immune responder cows have many beneficial qualities such as a lower occurrence of a number of common diseases (e.g. mastitis, ketosis, and metritis)^27–29^, better hoof health^30^, and increased total immunoglobulin and lactoferrin in colostrum compared to the average and low responders^26^. Since the heritability for HIR is moderately high (0.35), breeding for high immune response has been offered as a solution for reducing disease in dairy cattle^31^. Cartwright et al.^32^ recently demonstrated that BMCs from cows that were phenotyped as high immune responders based on their estimated breeding values (EBVs) produced more HSP70 and maintained cell proliferation better following heat stress treatment (4 h at 42 °C) compared to BMCs from cows phenotyped as average or low immune responders. Thus, high immune responder cows may also have a superior response to heat stress and global warming compared with their average and low herd-mates.

Epigenetic mechanisms, such as microRNAs (miRNAs), long non-coding RNAs (lncRNAs), DNA methylation, and histone modifications regulate gene expression^33^. The most extensively studied epigenetic modification is DNA methylation, which is best known for its role in genomic imprinting and X chromosome inactivation, and is integral to transcriptional regulation across entire genomes^34^. In mammals, DNA methylation usually occurs on the fifth carbon of cytosine, or 5-methylcytosine (meC), when it is followed by a guanine, to form a CG (or CpG) dinucleotide^35^. Depending on the location and context of DNA methylation in the genome it can up-regulate or suppress transcription^36,37^. DNA methylation of regulatory regions, such as promoters and enhancers, is typically associated with silencing of downstream genes^38–41^.

Two key studies established that calves born to cows who experienced stress (heat and transportation stress) during late gestation showed epigenetic changes to DNA methylation affecting genes associated with immune function, stress response, and cell signalling^42,43^. However, the mechanisms by which epigenetic regulation influences the acute response to heat stress in cattle remain largely unclear. Since genome regulation and the environment are bridged by epigenetic mechanisms, the aim of this study was to gain a better understanding of the epigenetic changes that occur in the bovine genome in response to a heat challenge and how these changes differ between cows of divergent immune response phenotypes.

## Materials and methods

### Animals

The dairy cattle used in this study were housed at the University of Guelph Elora Research Station. All cows were between the 3.5 and 4.2 years old, healthy, and not receiving any veterinary treatment at the time of this study. The average daily productions recorded were 90 kg (stdev = 15 kg) and 68 kg (stdev = 12 kg) for high and low cows, respectively. Two high and two low cows were in mid-lactation (100–199 days in milk) and the third high and third low cow were in late lactation (200 + days in milk, until dry off).

### Sample collection and BMC isolation

Sample collection was approved by Animal Care Services at the University of Guelph (Animal Utilization Protocol #3555) and all experiments were conducted in accordance with their rules and regulations. Blood was collected from healthy multiparous Holstein cows that had previously been classified as high (n = 3) or low (n = 3) immune responding animals based on their EBVs using the HIR technology. All blood collections were done during the winter months (November-March) to avoid previous exposure to heat and to ensure animals were not heat stressed. Whole blood was centrifuged at 1200×*g* for 20 min with the centrifuge break turned off. The buffy coat was isolated and diluted in a 1:2 ratio with PBS. The buffy coat-PBS solution was then layered over histopaque (Sigma-Aldrich, Oakville, ON) in a 1:1 ratio and spun at 1200×*g* for 10 min with the break off. The buffy coat was isolated and the volume was made to 25 ml with PBS. The cell suspension was spun at 150×*g* for 10 min with the brake on. Supernatant was discarded and cell pellet was resuspended in 3 ml PBS. The total number of cells per ml was determined using an ORFLO cell counter (ORFLO Technologies, Ketchum, ID). Cell viability was checked using trypan blue solution (Sigma-Aldrich, Oakville, ON.) via hemocytometer method.

### Heat treatment

Cells were diluted in RPMI media (Thermo-Fisher Scientific, Ottawa, ON) containing fetal bovine serum (Sigma Aldrich, Oakville, ON) and Penicillin–Streptomycin (Sigma Aldrich, Oakville, ON) to a concentration of 10^5^ viable cells/ml. Cells were then plated in three 1 ml replicates on a 24-well flat bottom cell culture plate (Sigma Aldrich, Oakville, ON) destined for TN treatment and three more 1 ml replicates on a second 24-well flat bottom plate for HS treatment. All plates were placed at 37 °C with 5% CO_2_ overnight. On the following day HS plates were moved to an incubator that was set at 42 °C with 5% CO_2_ for 4 h. TN plates remained at 37 °C for this period. Samples were collected immediately after completion of the 4-h heat challenge. For each plate, cell supernatant was collected from each well and the 3 wells for each cow were pooled. Adherent cells were lifted using Trypsin (Sigma Aldrich, Oakville, ON) and added to the pool before spinning at 300×*g*. Supernatant was discarded and the cell pellet was homogenized in 1 ml of Trizol reagent following the manufacturer’s protocol (https://tools.thermofisher.com/content/sfs/manuals/trizol_reagent.pdf.), flash frozen, and stored at − 80 °C.

The heat treatment method used in this study was done to assess molecular changes in response to acute heat stress. Previous studies have indicated changes in the concentration of heat shock proteins, including *HSP70* concentrations, following a heat stress treatment of one-two hours^44–47^. Furthermore, as part of a connected study, the concentration of HSP70 was measured in BMC cultures (n = 45), that included the animals that were used in this study, before and after the four-hour heat treatment using a commercial ELISA kit (Abclonal, Woburn, MA) following the manufacturer’s instructions (Supplementary Figure 1).

### DNA isolation

DNA was isolated from one control and one heat stressed (HS) sample from each of three high and three low cows, making a total of 12 samples (Table 1). The DNA was extracted following the manufacturer’s instructions for DNA extraction from Trizol Reagent (https://tools.thermofisher.com/content/sfs/manuals/trizol_reagent.pdf).

**Table 1:** Experimental design. This study contained a total of 12 samples from each of three cows immune response phenotyped as high (H) or low (L). A control (C) and heat stressed (HS) sample were generated for each cow, as indicated in the treatment column.

Sample	Cow	Phenotype	Treatment
1	1	H	C
2	1	H	HS
3	2	H	C
4	2	H	HS
5	3	H	C
6	3	H	HS
7	4	L	C
8	4	L	HS
9	5	L	C
10	5	L	HS
11	6	L	C
12	6	L	HS

### Library construction for DNA methylation analysis

Library construction was performed by Epigentek (Epigentek, NY) using their proprietary project workflow and methods as follows: for each sample, 40–50 ng of DNA was digested for 2 h with MSP1 at 20 U/sample at 37 °C, followed by 2 h with TaqI/sample at 65 °C. Digested DNA was size selected with < 300 bp DNA fragments and then bisulfite-treated with the Methylamp DNA Biulfite Conversion Kit (Epigentek Cat. # P-1001). Conversion efficiency of bisulfite treated DNA was determined by real time PCR using two primer pairs with control DNA. First primer pair is against bisulfite-converted DNA (β-actin) and the second primer pair is against unconverted DNA (GAPDH), for the same bisulfite-treated DNA sample. DNA was shown to be > 99% converted (Epigentek, NY).

A method of reduced representation bisulfite sequencing (RRBS), Enhanced MethylSeq (Epigentek, NY), was used to assess DNA methylation in control and HS samples. Libraries were prepared from 2–10 ng of genomic DNA using the PBAT parameter (Post-Bisulfite Adapter Tagging)^48^. Libraries were prepared by DNA end polishing and adaptor ligation followed by library amplification using indexed primers and library purification. Purified library DNA was eluted in 12 μl of water (Epigentek, NY).

### Enhanced MethylSeq sequence alignment and statistical analysis

Libraries were sequenced on an Illumina HiSeq 4000 with 50 bp single reads. After quality control of raw reads with FASTQC^49^ version 0.11.8, low quality and adapter trimming was performed using Trim Galore^50^ using the following parameters: minimum Sanger Phred score of 20, 3′ adapter trimming, and removal of reads shorter than 20 bp (RRBS-specific: additional 2 bps from any adapter-containing reads are removed. This option is used to avoid the possible filled-in cytosine after bisulfite treatment and restriction enzyme digestion).

Cleaned reads were aligned to the *Bos Taurus* reference genome (BosTau9) using Bismark^48^, version 0.203.0. Bismark uses Bowtie^51^, version 2.2.5 with the parameters “–directional” for targeted bisulfite sequencing libraries and “–pbat” for post-bisulfite prepared RRBS libraries. Samtools^52^, version 0.1.9, was used to sort the SAM files produced by Bismark and remove the duplicate reads resulting from PCR amplification. Methylation information was extracted from the final Bismark mapping result at the base resolution using the Bismark methylation extractor (version 0.203.0). A minimal read coverage of 5 and minimal quality score of 20 at each base position were applied. MethylKit^53^ was used to summarize methylation across promoter regions and conduct differential methylation analysis using default parameters. Promoters were defined as genomic regions from − 2000 bp to the transcription start site. Samples were filtered by coverage (minimum 5), normalized, merged (only promoter regions that were covered by a minimum of two samples in the comparison were included), and subjected to DMP identification with a cut off of 15% methylation difference and a q-value < 0.05. False discovery rate and multiple hypotheses testing were controlled using a Sliding Linear Model^54^.

The promoter region was selected for differential methylation analysis because DNA methylation in the promoter region is known to regulate gene activity: increased DNA methylation in the promoter region has been reported to suppress transcription^38,55^. Therefore, hypomethylation of a promoter region was assumed to suggest increased gene transcription whereas hypermethylation was assumed to indicate decreased transcription. The identified differentially methylated promoters (DMPs) were annotated against the RefSeq genes. Venn diagrams were generated with VENNY (version 2.1)^56^ to visualize overlapping gene sets.

### Functional analysis

The Ingenuity Pathway Analysis (IPA) package (QIAGEN Redwood City, http://www.qiagen.com/ingenuity) was used to perform metabolic pathway and gene network analyses using the gene sets associated with DMPs. Ensembl gene IDs were converted into human gene symbols with Biomart (http://www.biomart.org) before performing analyses in IPA^57,58^. Only the non-annotated genes, with a percentage of identity with the human homolog higher than 80%, were annotated by this approach^58,59^. Additionally, the Regulator Effects tool in the IPA package was used to identify potential regulators of the heat stress response in samples from high and low cows^58,59^.

## Results

### Overview of genome-wide DNA methylation profiling

In order to better understand the changes to DNA methylation resulting from heat stress and immune phenotype, we carried out Enhanced MethylSeq on BMCs from three high and three low immune responder dairy cows. Illumina HiSeq 4000 sequencing generated between 39.7 and 58.1 million reads per sample. After quality filtering, 72% to 81% of the reads were successfully aligned to the bovine reference genome (bosTau9). Of these reads, between 40 and 66% mapped uniquely across libraries. In total, we identified 9 to 22 million CpG sites per sample, of which 6.7% on average were methylated in the CpG context. Raw sequencing data and mapping statistics are summarized in Supplementary Table S1.

**Figure 1:**
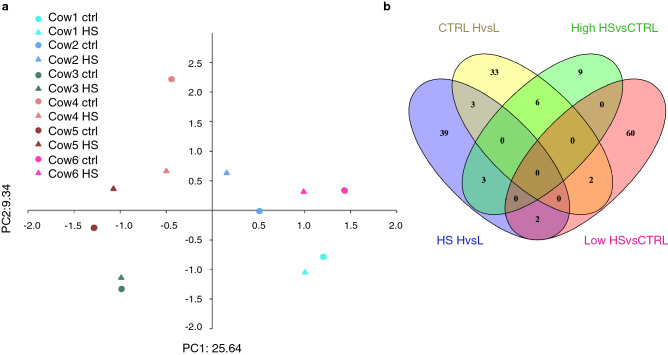
Effect of heat stress and immune response phenotype on DNA methylation in blood mononuclear cells from cows. (**a**) Principle component analysis showing overall methylation profiles across all samples. The first dimension explained 25.6% of variation. (**b**) Venn Diagram showing overlapping differentially methylated promoters from the four comparisons.

After correcting for multiple tests, differential DNA methylation analyses detected a total of 172 significant DMPs (q-value < 0.05) across the four comparisons described below. Principle component analysis separated the samples according to individuals indicating that individual differences were stronger than the effect of heat treatment on DNA methylation patterns (Fig. 1a). Some DMPs were shared across the four groups, but most were specific to the comparison (Fig. 1b).

### Differentially methylated promoters between immune phenotypes

#### Heat stressed samples

The comparison between HS samples from high cows and low immune responder cows (samples 2, 4, and 6 versus 8, 10, and 12, see Table 1) detected 47 significant DMPs, of which 34% were hypermethylated in high samples and 66% were hypomethylated in high samples. Five lncRNAs were among the 47 genes associated with the DMPs (see Supplementary Table S2).

The top hypermethylated promoter regions in HS samples from high immune responder cows were associated with the genes *OPRD1* (opioid receptor delta 1) and *NUDT15* (Nudix Hydrolase 15). The top hypomethylated promoter regions in samples from high immune responder cows were associated with the genes *BCL2L12* (BCL2 Like 12) and *CPEB1* (Cytoplasmic Polyadenylation Element Binding Protein 1). Additional genes associated with DMPs in this subset that were hypomethylated in samples from high cows include *HSPB9* (Heat Shock Protein Family B (Small) Member 9), *IL15* (Interleukin 15), and *NDRG1* (N-Myc Downstream Regulated 1) (see Supplementary Table S2).

#### Control samples

The comparison between control samples from high immune responder cows and low immune responder cows (samples 1, 3, and 5 versus 7, 9, and 11, see Table 1) revealed 44 DMPs, of which 43.5% were hypermethylated in high samples and 56.5% were hypomethylated in high samples. The top hypermethylated promoter regions in control samples from high immune responder cows were associated with the gene *SLC37A4* (Solute Carrier Family 37 Member 4) and an uncharacterized lncRNA. The top hypomethylated regions in high samples were associated with the genes *ALDOA* (Aldolase, Fructose-Bisphosphate A) and microRNA bta-mir2302. The promoter region of *IL15* was also hypomethylated in high samples in this subset (see Supplementary Table S3).

### Differentially methylated promoters between HS and control samples

#### High samples

The comparison between control and HS samples from high responder cows (samples 1, 3, and 5 versus 2, 4, and 6, see Table 1) revealed 18 DMPs, of which just over half were hypermethylated in HS samples (55.5%). One lncRNA is included in these 18 genes (see Supplementary Table S4).

The top hypermethylated promoter regions in HS samples were associated with the genes *ARSG* (Arylsulfatase G), *APC2* (APC Regulator of WNT Signalling Pathway 2), and an uncharacterized lncRNA. The top hypomethylated promoter regions in HS samples were associated with the genes *IGF2* (Insulin Like Growth Factor 2) and *CPEB1* (Cytoplasmic Polyadenylation Element Binding Protein 1). *JMJD8* (Jumonji domain-containing protein 8) was also hypomethylated in HS samples (see Supplementary Table S4).

#### Low samples

The comparison between control and HS samples from low immune responder cows (samples 7, 9, and 11 versus 8, 10, and 12, see Table 1) detected 64 DMPs, of which 53% were hypermethylated HS samples and 47% were hypomethylated in HS samples. Among these 64 DMPs were six lncRNAs (see Supplementary Table S5).

In HS samples, the top hypermethylated promoter regions were associated with the genes *RHOT2* (Ras Homolog Family Member T2) and *NCKIPSD* (NCK Interacting Protein With SH4 Domain), and top hypomethylated promoters were associated with the genes *MMTAG2* (Multiple Myeloma Tumor-Associated Protein 2 Homolog), *CCDC40* (Coiled-Coil Domain Containing 40), and *NGF* (Nerve Growth Factor) (see Supplementary Table S5).

### Metabolic pathways and gene networks connecting DMPs

**Figure 2:**
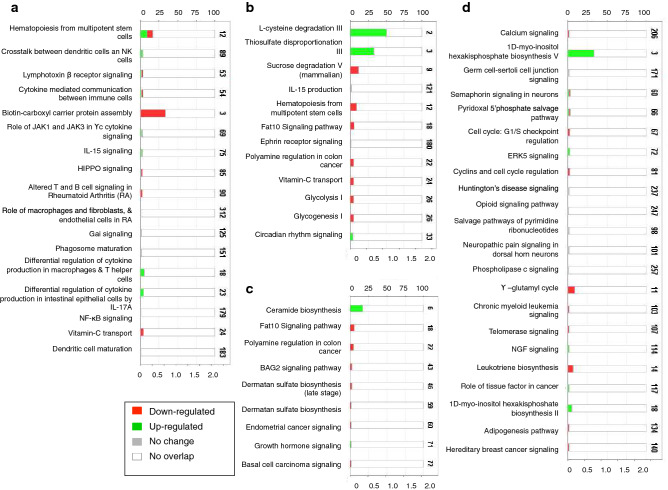
Canonical pathways identified as significantly enriched using IPA in (**a**) High versus low heat stressed samples, (**b**) high versus low control heat stressed samples, (**c**) control versus heat stressed high samples, (**d**) control versus heat stressed low samples. The bottom axes of graphs shows the − log(p-value), and the upper axes show the percentage of genes in each pathway. The total number of genes in each pathway is shown at the end of each bar. This figure was generated through the use of IPA (QIAGEN Inc., https://www.qiagenbioinformatics.com/products/ingenuitypathway-analysis).

Genes associated with DMPs were mapped to the IPA database for metabolic pathway and gene network exploration. Significantly enriched metabolic pathways were identified in all four sets of DMPs. However, this result should be interpreted with caution because pathways were generally represented by a small number of genes^58^. The top canonical pathways associated with DMPs in heat stressed high versus low immune responder cows included *Hematopoiesis from multipotent stem cells* (p = 2.07E−04), *Crosstalk Between Dendritic Cells and Natural Killer Cells* (p = 5.55E−04), *Lymphotoxin Beta Receptor Signalling* (p = 4.12E−03), and *Role of Cytokines in Mediating Communication Between Immune Cells* (p = 4.28E−03) (Fig. 2a). In control samples, the top canonical pathways associated with DMPs in high versus low samples included *IL15 Production* (p = 1.33E−02) (Fig. 2b).

The DMPs identified within high and low immune responder subsets of heat stressed versus control samples were associated with various signaling pathways and apoptosis. The top canonical pathway associated with DMPs in high immune responder cows was *Ceramide Biosynthesis* (p = 4.22E−03) (Fig. 2c). In low immune responder cows, the top scoring canonical pathway was calcium signaling (p = 1.78E−03), which included four genes [*GRIN1* (Glutamate Ionotropic Receptor NMDA Type Subunit 1), *HDAC10* (Histone Deacetylase 10), *HDAC4* (Histone Dacetylase 4), and *PNCK* (Pregnancy Up-Regulated Nonubiquitous CaM Kinase)] (Fig. 2d).

**Figure 3:**
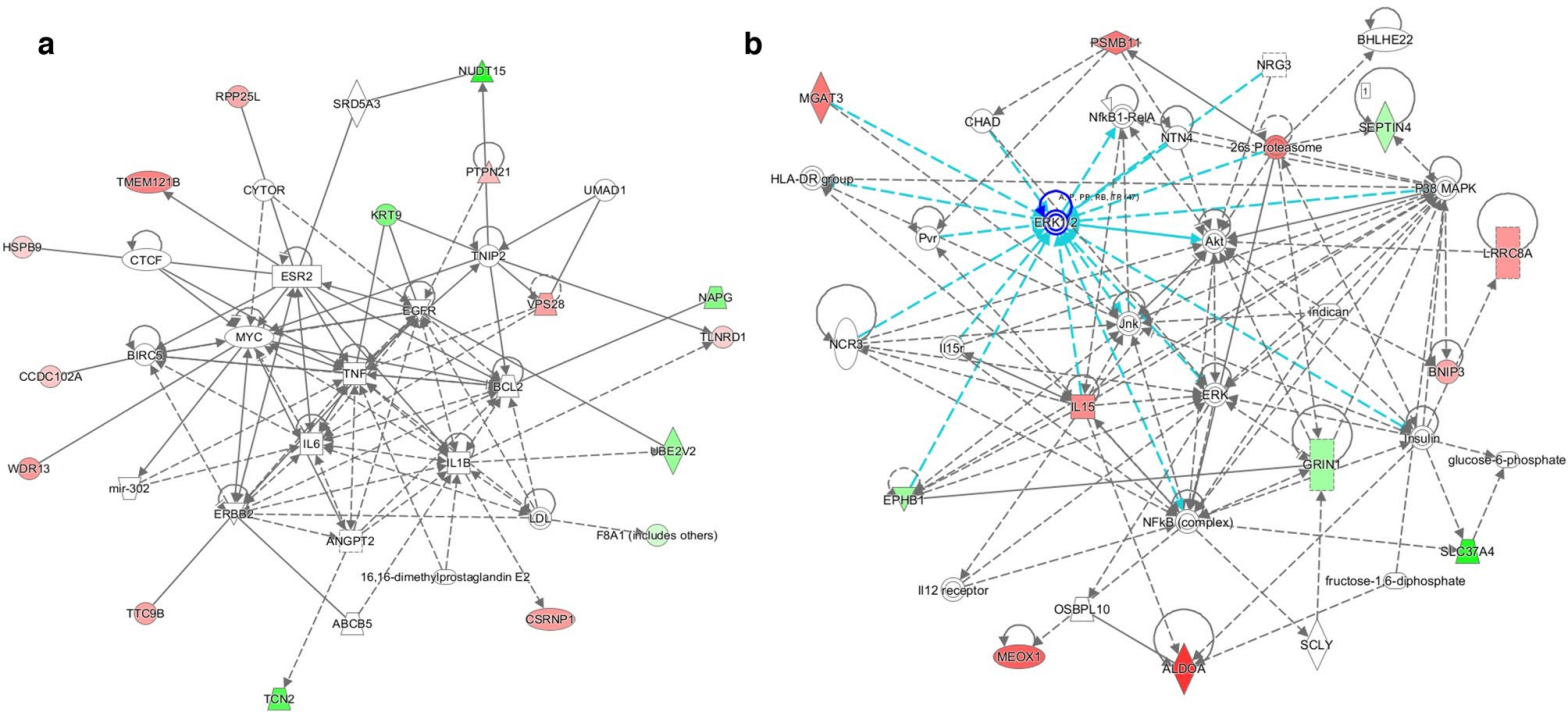
The top-scoring regulatory networks identified using IPA software connecting DMPs between (**a**) high and low heat stressed samples was *Cell-to-cell Signalling and Interaction, Amino Acid Metabolism, Cell Death and Survival*, and between (**b**) high and low control samples was *Immunological Disease, Neurological Disease, Skeletal and Muscular Disorders*. Genes that had hypermethylated and hypomethylated promoter regions in high samples are green and red respectively. Solid and dashed lines between genes represent known direct and indirect gene interactions, respectively. The shapes of the nodes reflect the functional class of each gene product: transcriptional regulator (horizontal ellipse), enzyme (vertical rhombus), cytokine (square), phosphatase (triangle), kinase (inverted triangle), complex/other (circle), transporter (trapezoid), and enzyme (rhombus). The networks were generated through the use of IPA (QIAGEN Inc., https://www.qiagenbioinformatics.com/products/ingenuitypathway-analysis).

The DMPs were also grouped in gene regulatory networks with the IPA software. The top scoring regulatory network connecting DMPs identified in high versus low heat stressed samples was *Cell-to-cell Signalling and Interaction, Amino Acid Metabolism, Cell Death and Survival* (Fig. 3a). The top network connecting DMPs in high versus low control samples was *Neurological Disease, Skeletal and Muscular Disorders, Immunological Disease* (Fig. 3b). The top network connecting DMPs in control versus HS samples from high cows was *Cancer, Gastrointestinal Disease, Organismal Injury and Abnormalities*. The top network connecting DMPs in control versus HS samples from low cows was *Carbohydrate Metabolism, Organismal Development, and Cell Morphology.*

**Figure 4:**
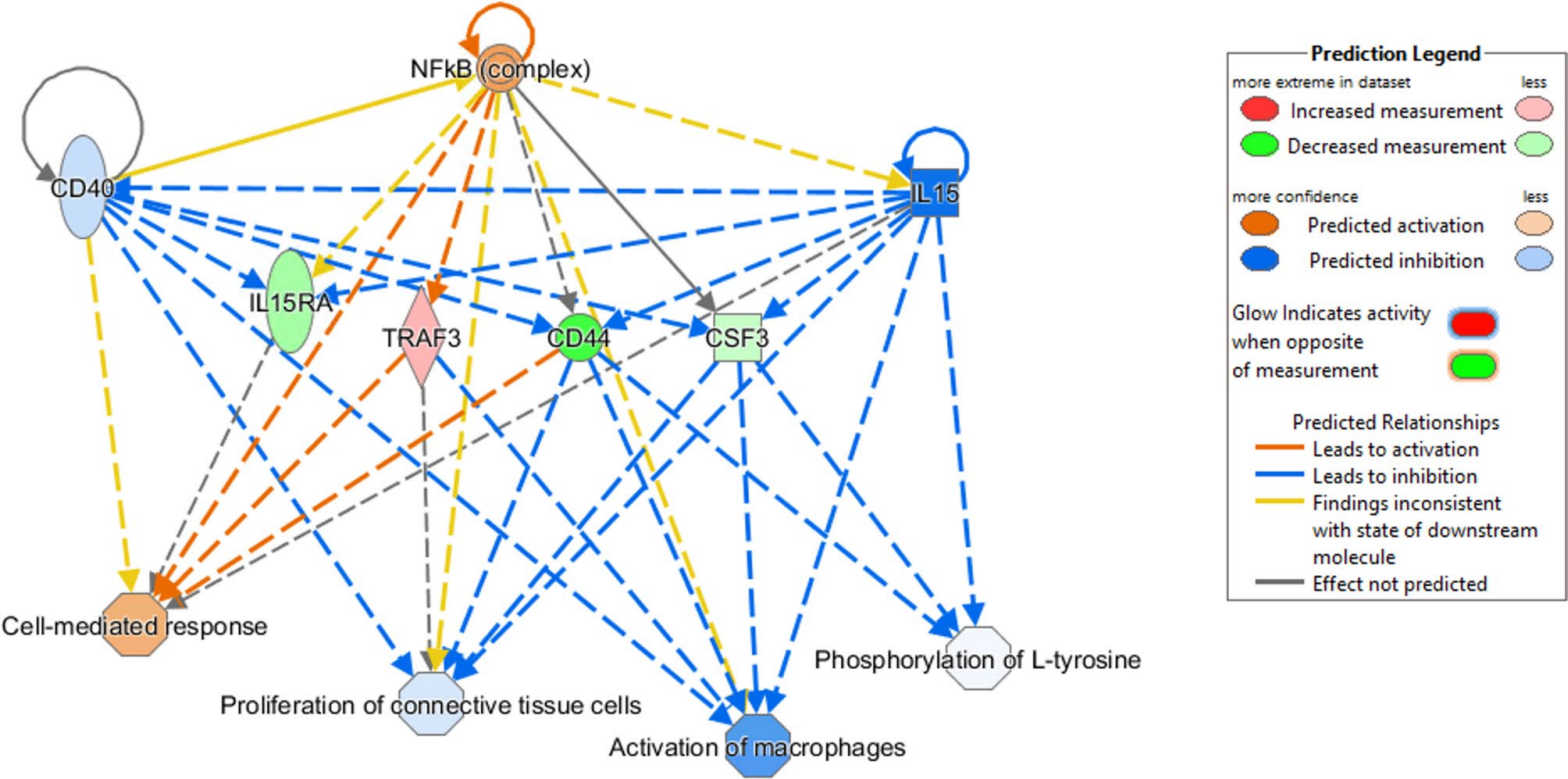
Upstream regulators of the networks of genes with DMPs identified between high and low heat stressed samples. In the upper tier, there are three predicted upstream regulators (*IL15*, *CD40*, and NF-κB). The shapes of the nodes reflect the functional class of each gene product: enzyme (vertical rhombus), transcription regulator (vertical ellipse), cytokine (square), and complex/group/other (circle). In the lower tier, the expected phenotypic consequences of changes in gene expression are shown using the Ingenuity Knowledge Base (absolute z-score > 2 and p-value < 0.05). The octagonal symbol defines function, solid and dashed lines between genes represent known direct and indirect gene interactions, respectively. Orange leads to activation, while blue leads to inhibition predicted relationships. The networks and regulator effects were generated through the use of IPA (QIAGEN Inc., https://www.qiagenbioinformatics.com/products/ingenuitypathway-analysis).

The Regulator Effects tool in the IPA package was used to identify potential regulators of the heat stress response in samples from high and low cows. This tool integrates upstream regulator results with downstream effects results to build causal hypotheses that help to interpret what may be occurring upstream to cause particular phenotypic or functional outcomes. Three regulators were identified [*NFkB* (Nuclear Factor-kB), *IL15*, and *CD40* (Cluster of Differentiation 40) that are involved in *Cell-mediated response*, *Activation of macrophage*, and *Proliferation of connective tissue cells* (*IL15RA* (IL15 Receptor Subunit Alpha), *TRAF3* (TNF Receptor Associated Factor 3), *CD44* (Cluster of Differentiation 44), and *CSF3* (Colony Stimulating Factor 3)] (Fig. 4).

## Discussion

Heat stress (HS) negatively affects production^60,61^ and suppresses immune^62,63^ and reproductive function in bovines^63^. There is evidence that HS increases mastitis incidence^64^ and other health problems including ketosis, liver lipidosis, and even mortality^64,65^. Studies investigating the molecular changes in response to heat stress have shown epigenetic and transcriptional modulation of genomic regions associated with immune function, stress response, metabolism, and cell signalling^42,43,66^. To our knowledge, this is the first attempt made to study genome-wide DNA methylation changes to promoter regions resulting from heat stress in a population of BMCs isolated from dairy cows of distinct immune response phenotypes.

Analysis of differential methylation of promoter regions by principle component analysis revealed that the most variability in promoter regions was between individuals. However, 172 significant DMPs (q-value < 0.05) were identified across the four comparisons discussed below. Indeed, results from this study suggest that high cows may also have increased expression of interleukin 15 (*IL15*): within the HS and control groups, the *IL15* promoter region was significantly hypomethylated in samples from high cows compared to low cows (see Supplementary Tables S2 and S3). Indeed, increased DNA methylation within gene promoter regions has been reported to suppress transcription^39^, suggesting that hypomethylation of gene promoter regions could be associated with up-regulated gene expression. *IL15* is important for proliferation, survival, and differentiation of natural killer (NK) cells and T cells, and is implicated in the signalling pathway categories for apoptosis, cellular immune response, and cytokine signalling. The *IL15* gene plays a key role in the immune response to viral and bacterial infections by regulating cells of both the innate and adaptive immune system^67^. Previous work has shown that *IL15* and its receptor, *IL15Ra*, support NK cell homeostasis under resting conditions, and mediate NK cell survival and differentiation into functional NK cells capable of killing virally infected cells^68^. As such, up-regulation of IL15 expression in BMCs could contribute to the superior immune response phenotype of high immune responder cows by enhancing the function of their immune cells. Further expression studies to investigate this potential difference in *IL15* between high and low cows will be informative.

The analysis of individual DMPs across the bovine genome revealed genes involved in a range of functions. Within the HS groups, signalling pathways associated with DMPs between high and low samples included cytokine communication, cell death and survival, and cell-to-cell signalling and interaction. Many of the DMPs identified in this comparison were associated with genes that are important for protecting cells against stress. For example, the gene *BCL2L12* is an anti-apoptotic member of the Bcl2 protein family^69^. Out of all of the DMPs in heat stressed high immune responder versus low immune responder samples, the promoter region of *BCL2L12* showed the lowest methylation density in high samples (Table S2). Higher expression of *BCL1L12* in high immune responder cows could be related to more anti-apoptotic activity and better cell survival. Furthermore, *HSP70* has been show to interact with and protect *BCL2L12* from degradation^70^, supporting the protective profile of DMPs in HS samples from high cows. Indeed, heat shock proteins have been extensively studied for their role in protecting cells from heat and other forms of stress (reviewed in^71^). These molecular chaperones have cryoprotective and protein re-folding functions that preserve protein structure and transport^8^ and therefore, elevated expression of HSPs is a desired response to an increase in body temperature^9–11^. The cytoprotective effect of hsp70 is partly due to its ability to impede apoptosis^72^. As a means of promoting cell survival, the heat stress response and HSPs are also known to play a role in inflammatory signalling by regulating the production of inflammatory cytokines^72^.

Additional genes with protective functions whose promoters were hypomethylated in BMCs from high cows include heat shock protein B9 (*HSPB9*) and N-myc downstream-regulated gene 1 (*NDRG1*) (Table S2). Expression of *HSPB9* in response to heat stress has been shown in brain, liver, and muscle tissue from chicken^73^. The *NDRG1* gene is an important stress response protein that responds to a variety of cellular stressors and has a putative function in suppression of tumour metastasis^74,75^. The *NDRG1* gene is also induced by hypoxia and iron depletion^76^.

The top scoring regulatory network connecting genes with DMPs between high and low HS samples was *Cell-to-cell Signalling and Interaction, Amino Acid Metabolism, Cell Death and Survival* (Fig. 3a). The tumour necrosis factor (*TNF*) molecule occupies a central position in the network shown in Fig. 3a. The *TNF* gene has a broad range of functions, including cell proliferation and differentiation, inflammation, and apoptosis^77^. *TNF* is also part of the *IL-1* and *IL-6* fever cascade that acts on the hypothalamus during infection with certain pathogens^78^. This network may represent the dual effect of HSPs on cell survival—on the one hand, as discussed here, HSPs play a critical role in cell survival in response to heat stress. However, it has also been shown that HSPs sensitize cells to certain apoptotic stimuli, such as *TNF*. Ran et al.^79^ showed that *HSP70* enhances TNF-mediated apoptosis by binding to *IκB kinase* γ and impairing *NF-κB* survival signalling. However, a study by Imao et al.^80^ demonstrated that repeated heat stress was needed to initiate this apoptotic pathway.

The top network connecting DMPs in high versus low control samples (Fig. 3b) was *Immunological Disease, Neurological Disease, and Skeletal and Muscular Disorders*. The network shown in Fig. 3b shows groups and complexes important for immune function occupying central positions and having direct connections with genes identified with DMPs. For example, Jun N-terminal kinases (JNKs) and p38 mitogen-activated protein kinases (MAPKs) have important roles in the cellular response to many types of stress, as well as regulating the activity of inflammatory mediators^80^. Also in a central position was *NF-κB*, which is critical for regulating immune function^81^. The Regulatory Effects tool in IPA also identified *NF-κB*, along with *IL15*, and *CD40*, as regulators of four genes (*IL15RA*, *TRAF3*, *CD44*, and *CSF3*) with DMPs between high and low HS samples (Fig. 4). The NF-κB complex functions as a transcription factor to regulate a broad range of biological processes including immune function, inflammation, and stress responses^79^. These regulators may represent some of the differences in immune function between high and low cows and further study could reveal molecular differences between immune response phenotypes.

The network shown in Fig. 3b also shows the V-akt murine thymoma viral oncogene homologue molecule (*AKT*) in a central position, having connections with genes identified with DMPs (for example, *IL15* and *BNIP3*). The *AKT* gene is a main regulator of glucose homeostasis^82^, suggesting that high and low cows could have differences in this process. Indeed, the promoter region of the solute carrier family 36 member 4 gene (*SLC37A4*), better known as the glucose-6-phosphate transporter (*G6PT*), had the highest density of methylation (hypermethylated) in high samples compared to lows. This sugar-phosphate exchanger maintains glucose homeostasis and neutrophil energy homeostasis, and deficiency is responsible for glycogen storage disease type Ib^83^. Hypermethylation of the *G6PT* promoter region in BMCs from high cows could point to decreased expression compared to low cows under resting conditions. Further expression profile studies should be conducted to investigate this potential difference in energy metabolism between high and low cows.

Significant DMPs were also identified in the comparison between control and HS treatment for both high and low samples. However, the specific DMPs that were identified in this comparison differed between high and low groups, suggesting that the cellular response to HS is different in cows of distinct immune response phenotypes. In samples from high cows, the top up-regulated metabolic pathway associated with the DMPs identified between control and HS samples was *Ceramide Biosynthesis* (Fig. 2c); ceramide plays a role in mediating apoptosis in response to cytokines and environmental stress. The promoters with the lowest methylation density in HS samples were insulin-like growth factor (*IGF2*) and cytoplasmic polyadenylation element-binding protein (*CPEB1*). *CPEB1* is a key factor in controlling mRNA translation. Xiaoping et al.^84^ demonstrated that hypomethylation of the *CPEB1* promoter resulted in overexpression, suggesting that hypomethylation of the promoter region in our study could also result increased expression.

The jumonji C domain-containing protein *JMJD8* gene promoter was hypomethylated in HS compared to control samples from high cows. Jumonji proteins have been shown to regulate cellular processes by hydroxylating or demethylating histone and non-histone targets^85^. An important function of genes in the jumonji family is modulation of gene expression via histone post-translational modifications^86^. The *JMJD8* gene is involved in angiogenesis and cellular metabolism^87^, and is also a positive regulator or TNF-induced NF-κB signalling pathway^88^. Mass spectrometry analysis revealed two HSP proteins bound to *JMJD8*: *HSPA5* and *HSP90B1*^89^, suggesting that *JMJD8* could play a role in the stress response. A previous study demonstrated a role for another jumonji family protein (*JMJD1A*) in regulating the response to cold stress (4 °C for one week) in mice by up-regulating genes associated with the thermogenic response in brown adipose tissue^90^.

Eight DMPs were hypermethylated in HS samples from high cows, including *APC2* and *BNIP3*. *APC2* is a regulator of the WNT signalling pathway and *BNIP3* plays a critical role in inducting autophagy during heat stress^91^ and is also proapoptotic^92^. The hypermethylation of the *BNIP3* gene promoter in HS samples from high cows suggests decreased mRNA expression compared to control samples, potentially increasing cell survival during heat stress.

The comparison between control and HS samples from low cows revealed 64 DMPs associated with a range of metabolic and signalling pathways. The promoter region with the lowest methylation density (hypomethylated) in HS samples was the multiple myeloma tumour-associated protein 2 (*MMTAG2*) gene. Luo et al.^93^ showed that *MMTAG2* (also known as C1orf35) is involved in cell growth by promoting cell cycle progression from G1 to S. Overexpression of *MMTAG2* results in up-regulation of c-MYC and subsequent accelerated cell proliferation^94^, perhaps revealing a cell survival mechanism activated in low cows. The promoter regions of two histone deacetylases (*HDAC10*, *HDAC4*) were hypermethylated in HS samples from low cows. In general, acetylation of histones is permissive to gene expression because it opens up chromatin so that it is accessible to transcription factors, whereas deacetylation represses gene expression. Previous studies have shown that expression of HSPs, including *HSP70*^95,96^, increased as a result of hyperacetylation via inhibiting the expression of histone deacetylases. Indeed, Fritah et al.^97^ demonstrated that heat-shock factor 1 (a transcription factor that activates HSPs), is a master regulator of chromatin acetylation across the genome in response to heat stress. Hypomethylation of the promoter regions of both *HDAC4* and *HDAC10* in HS samples suggests that expression of these genes was decreased upon heat stress. Hence, a mechanism of cell protection used by BMCs from low cows could be activating expression of HSPs via decreasing expression of histone deacetylases.

## Conclusion

We identified significant DMPs in BMCs isolated from immune phenotyped Holstein dairy cows, identified as high or low immune responders, under both control and heat stress conditions. These results suggest that DNA methylation of promoter regions could contribute to variation in immune phenotypes in dairy cows, and also variation in the response to heat stress. The DMPs differed between samples from high and low cows, but our results revealed pathways that could provide protection to the cells during heat stress in both immune phenotypes. In samples from high cows, heat stress resulted in differential methylation of gene promoters associated with stress response and apoptosis prevention, whereas in low cows, heat stress affected promoter methylation of genes associated with cell proliferation and histone deacetylases. Our results also revealed potential differences between high and low cows under control conditions: hypomethylation of the *IL15* promoter region in samples from high cows suggests higher expression of this cytokine in high cows compared to their low herd mates. Additionally, hypermethylation of the *G6PT* promoter in samples from high cows could point to lower mRNA expression compared to low cows, perhaps revealing a difference in energy metabolism.

Future studies evaluating the transcription profile of genes in response to heat stress will provide more insight into the functional relevance of our findings. Furthermore, in addition to the changes in DNA methylation of promoter regions, it is likely that heat stress alters gene expression through other epigenetic modifications as well, such as histone modifications and microRNAs. More information about changes in the epigenomic landscape in response to heat stress as well as corresponding transcriptional changes would improve our understanding of the molecular differences between immune phenotypes in bovine. This knowledge will allow us to better understand the relationship between immune response phenotype and response to heat stress. Considering the moderately high heritability of HIR (0.35)^31^, immuno-genetics could provide a means to genetically select for improved response to heat stress. Identifying cattle with both genetically enhanced immunity and resilience to heat stress would provide a new tool to increase livestock efficiency in the context of climate change.

## Supplementary Information

Supplementary Figure 1.

Supplementary Tables.

## Data Availability

The datasets generated and analysed during this study are available in the GEO repository with the accession number GSE163222.
