# Supplementary material for: Heat stress and immune response phenotype affect DNA methylation in blood mononuclear cells from Holstein dairy cows
Source: Sci Rep. 2021 May 31;11:11371. doi: 10.1038/s41598-021-89951-5 (PMC8166884; doi:10.1038/s41598-021-89951-5)
Supplement: Supplementary file 1 — Supplementary Figure 1. [file 41598_2021_89951_MOESM1_ESM.pdf]

Heat stress and immune response phenotype affect DNA methylation in blood mononuclear cells from Holstein dairy cows

Livernois AM[1,2]*, Mallard BA[1,2], Cartwright SL[1], Cánovas A[2]
[1]Dept of Pathobiology, Ontario Veterinary College, University of Guelph, Guelph, ON
[2]Centre for Genetic Improvement of Livestock, Dept. of Animal Biosciences, University of Guelph, Guelph, ON

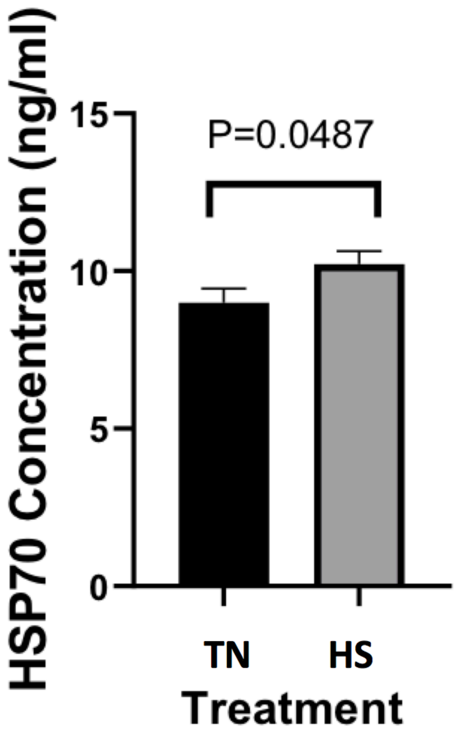

Supplementary Figure 1. HSP70 concentration for all immune response phenotypes following a four-hour incubation in either a temperature neutral (TN) environment (control) or a heat stress environment (HS).
